# Supplementary material for: Cu Nanoparticles Modified Step-Scheme Cu2O/WO3 Heterojunction Nanoflakes for Visible-Light-Driven Conversion of CO2 to CH4
Source: Nanomaterials (Basel). 2022 Jul 2;12(13):2284. doi: 10.3390/nano12132284 (PMC9268155; doi:10.3390/nano12132284)
Supplement: Supplementary file 1 [file nanomaterials-12-02284-s001.zip › nanomaterials-1794895-supplementary.pdf]

nanomaterials

Supplementary Material

Cu Nanoparticles Modified Step-Scheme Cu_2O/WO_3 Heterojunction Nanoflakes for Visible-Light-Driven Conversion of CO_2 to CH_4

Weina Shi [1], Ji-Chao Wang [2,3,*], Aimin Chen [1], Xin Xu [1], Shuai Wang [1], Renlong Li [2], Wanqing Zhang [2] and Yuxia Hou [2,*]

[1] School of Chemistry and Materials Engineering, Xinxiang University, Xinxiang 453000, China; shiweina516@163.com (W.S.); chenaimin1978@163.com (A.C.); xuxin202111@163.com (X.X.); wangshuai00129@163.com (S.W.)
[2] College of Chemistry and Chemical Engineering, Henan Institute of Science and Technology, Xinxiang 453000, China; rlli@hist.edu.cn (R.L.); zhangwqzzu@163.com (W.Z.)
[3] College of Chemistry, Zhengzhou University, Zhengzhou 450000, China
* Correspondence: wangjichao@hist.edu.cn (J.-C.W.); yxhou@163.com (Y.H.); Tel.: +86-0373-304-0418 (J.-C.W. & Y.H.)

S1. Catalytic Experiment for CO_2 Photoreduction

Photocatalytic activity for CO_2 reduction with water vapor was evaluated in a stainless-steel cylindrical vessel with a length of 15 cm and a volume of 180 mL, and the light source was a 300 W Xenon arc lamp (PLS-SXE 300, Beijing Trust tech Co. Ltd., China) with a UV cutoff filter ($\lambda > 400$ nm). The catalyst was put on a glass sheet (1.8 × 2 cm) equipped with a quartz window. Before lighting, the reaction setup was vacuum-treated, and then high purity CO_2 gas (99.995%) was purged into the reaction system several times. The compressed high purity CO_2 gas passed through a water bubbler to generate a mixture of CO_2 and H_2O vapor. After illumination, the gaseous products were quantifiably identified by offline analysis using a GC-7890IIgas chromatograph (Techcomp Corp., China) equipped with FID and TCD detectors, and the columns were TDX-01 and Porapak-Q, respectively. Then, the hydrocarbon product was further analyzed by GC-MS (Agilent 7890A-5975C) equipped with a DB-FFAP capillary column. In order to evaluate the stability of the synthesized composites, the $Cu/Cu_2O/WO_3$ model sample was refreshed by electrochemical treatment before washing and drying, and its photocatalytic performance was reassessed.

Table S1. ICP-AES analysis of Cu_2O/WO_3 and $Cu/Cu_2O/WO_3$ samples.

Sample	Ratio of Cu/W atoms (%)
WO_3	0.00
Cu_2O/WO_3	16.66
$Cu/Cu_2O/WO_3$	18.22
Cu/Cu_2O	100
Cu_2O	100

Table S2. Band gaps of pure WO_3, Cu_2O, Cu_2O/WO_3 and $Cu/Cu_2O/WO_3$ samples.

Sample	Band gap(eV)
WO_3	2.83
Cu_2O/WO_3	2.32*
$Cu/Cu_2O/WO_3$	2.09*
Cu_2O	2.03

* The above value was apparent band gap of the sample.

Table S3. Comparison of performance CO_2 conversion of the obtained material with other reported catalysts.

Catalyst	Experimental Conduction	Performance	Reference
$Cu/Cu_2O/g\text{-}C_3N_4$	300W Xe lamp	CO: 10.8 μmol/g_{cat}/h<br>CH_4: 3.1 μmol/g_{cat}/h<br>CH_4/CO: 0.29	Carbon, 2022, 193, 272-284.
$Cu/CuO_x/TiO_2$	56 mW/cm^2 LED (425 nm)	CO: 0.7 μmol/g_{cat}/h<br>CH_4: 1.2 μmol/g_{cat}/h<br>CH_4/CO: 1.71	Nanomaterials, 2022, 12, 1584.
$Cu\text{-}Ti_3C_2Tx/g\text{-}C_3N_4$	300W Xe lamp (170 mW/cm^2, 400nm cutoff filter)	CO: 1225.5 μmol/g_{cat}/h<br>CH_4: 90 μmol/g_{cat}/h<br>CH_4/CO: 0.07	Chem. Eng. J., 2022, 446, 137028.
1%CuO_x/TiO_2(101)	300 W Xe lamp	CO: 0.32 μmol/g_{cat}/h<br>CH_4: 2.29 μmol/g_{cat}/h<br>CH_4/CO: 7.15<br>H_2: 2.96 μmol/g_{cat}/h	Appl. Surf. Sci., 2021, 564, 150407.
$Cu_2O/Ti_3C_2T_x$	300 W Xe lamp	CO: 17.55 μmol/g_{cat}/h<br>CH_4: 0.96 μmol/g_{cat}/h<br>CH_4/CO: 0.05	Appl. Surf. Sci., 2021, 542, 148685.
$Cu_2O@Cu_3(BTC)_2$	500 W Xe lamp (400nm cutoff filter), Thin film on copper mesh	CO: 0.73μmol for 8h	Angew. Chem. Int. Ed., 2021, 6, 8455-8459.
$Cu/Cu_2O/WO_3$	300 W Xe lamp (170 mW/cm^2, 400nm cutoff filter,)	After 24 h illumination<br>CH_4: 101.2 μmol/g_{cat}/h<br>H_2: 13.3 μmol/g_{cat}/h<br>O_2: 143.7 μmol/g_{cat}/h	This work

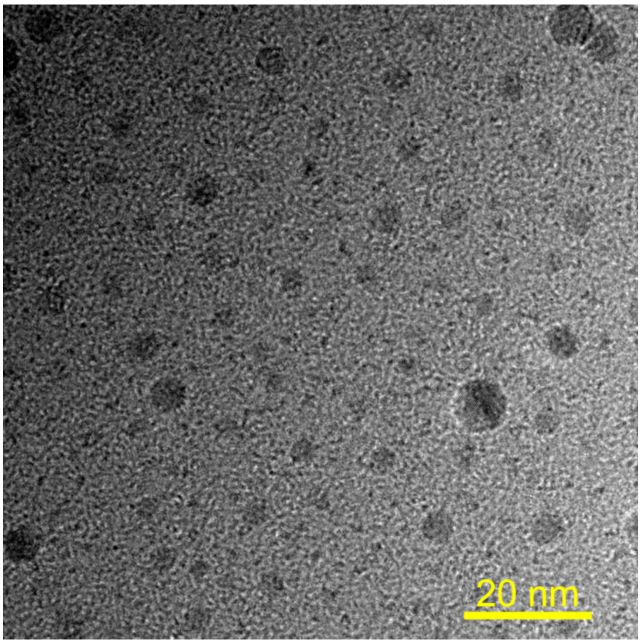

Figure S1. TEM image of Cu nanoparticles in $Cu/Cu_2O/WO_3$ sample.

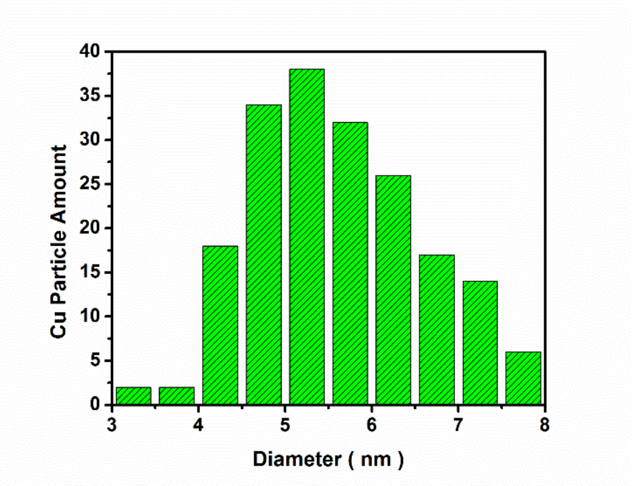

Figure S2. Size distribution histogram of Cu nanoparticles.

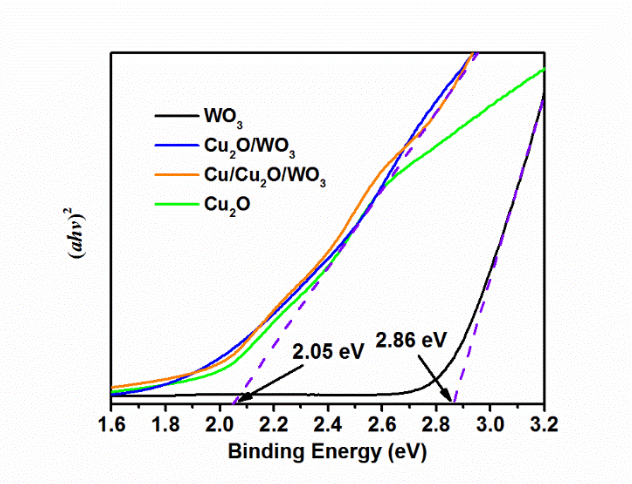

Figure S3. The curves of $(\alpha h\nu)^2$ versus photo energy ($h\nu$).

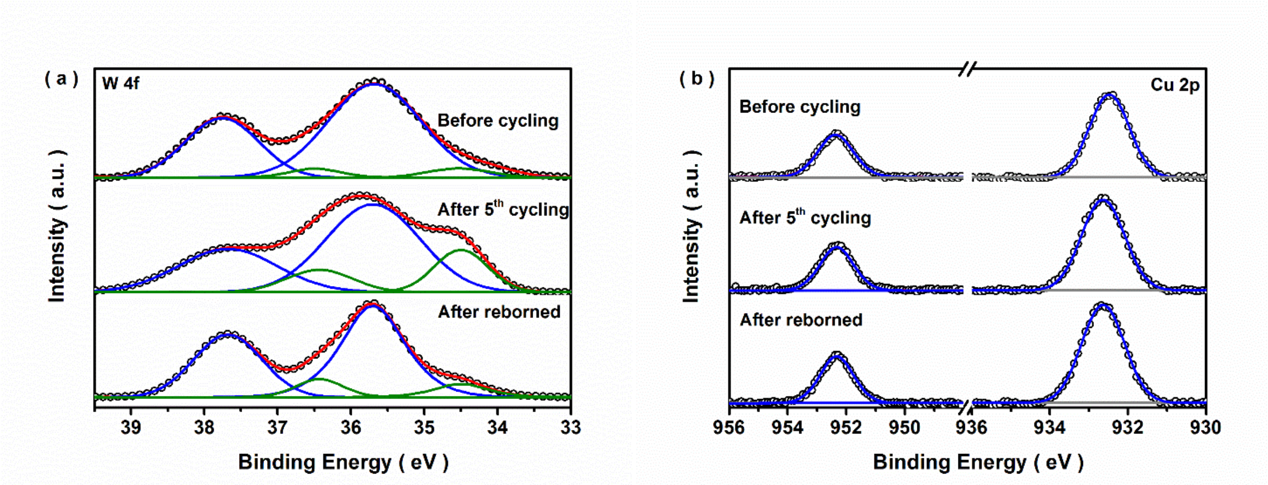

Figure S4. XPS spectra of $Cu/Cu_2O/WO_3$ in the cycling tests **(a)** W 4f and **(b)** Cu 2p.
